# The Mind Symptoms of Our Homœpathic Drugs

**Published:** 1895-10

**Authors:** J. R. Haynes

**Affiliations:** Indianapolis, Ind.


### THE MIND SYMPTOMS OF OUR HOMŒOPATHIC DRUGS.

J. R. HAYNES, M. D., INDIANAPOLIS, IND.

Hahnemann tells us that we ought to give the mind symptoms a prominent position in the totality when taking a case—*i. e.*, that they are of the greatest importance in selecting the proper curative remedy for the case. This I have found time and again to be true, and that it should never be neglected at any time.

Let us illustrate: We are called upon to prescribe for a case; we find a dull, heavy headache through the whole top of the head, somewhat aggravated by motion; nose stuffed up or running; tongue heavily coated white; clammy, sweetish, sticky taste in the mouth; throat sore, painful upon swallowing; no appetite, disgusted with the sight or thought of food; considerable thirst, wants large quantity when drinking; gone sensation in the stomach and abdomen; wants to urinate often, but small quantities at a time, but must go at once—somewhat painful in passing at the time; soreness of the muscles of the limbs and whole body, worse by light pressure than by hard, but no particular pain upon motion; spasmodic, hoarse cough, which cannot be controlled until a small quantity of mucus is raised; skin hot and dry; feels the least draft of air; better when heated up, and when perspiring wants to keep up the perspiration, as all of the symptoms are better.

Now, every one of these symptoms is found in the pathogenesis of Mercurius-viv.; shall we give it?

Let us refer to the mind symptoms of this case and see if they will correspond: Weak, languid, no ambition; gets set down it is hard work to move; has to use great energy to stir; gloomy, sad, and despondent; feels as if all friends had forsaken them; thinks that every one is talking about or making fun of them; gets angry at the least trifles, then tears will run down their cheeks; every little thing annoys them; wishes they were dead and out of the way.

Mercurius-viv. has some of these symptoms, but not the most prominent ones. Shall we give it here? If we do we will most certainly meet with a failure, because it does not have the totality of the mind symptoms of the case.

Again, we have a severe headache over the whole head, dizzy through the forehead, aggravated by motion or on attempting to raise up; tongue coated yellowish brown on the posterior portion, red and somewhat pointed tip, sore stinging, burning fauces; soreness through the larynx; strangling tickling in the throat-pit, causing severe coughing spells; nose somewhat stuffed up or discharging sticky mucus; no appetite; dry, sticky taste in the mouth; thirsty, but takes moderate drinks; sticky, nauseating, sweetish mucus in the fauces; griping, cutting pains in the stomach and bowels, with flatulent colic; diarrhœaic stools; pains relieved for a time after stool, but soon returning again; urine scant and reddish brown, with sediment upon standing, with decomposed odor; joints swollen and painful, causing great restlessness; pains somewhat relieved by motion; must move and turn about; cannot sleep until after midnight, and when tired out drops to sleep, which is restless, and when awakened feels perfectly exhausted and cross.

Here we have a perfect picture of the symptoms of Rhus-tox.

Let us look at the mind symptons. Wakes up cross and snappish; cannot bear to be spoken to; can hardly give a civil answer; wants to be let alone, yet wants attendants where they can be seen; feels afraid to be left alone. Now if we give Rhus in this case we most certainly meet with a failure, as the totality of mind symptoms does not come under the head of that remedy, and cannot cure them.

Again, we have a patient who has a dull, heavy headache, dizzy through the forehead, aggravated by motion; attacks of vertigo, especially when attempting to walk; reels and staggers as if intoxicated; tongue heavily coated white and flabby; gives impression of the teeth; sticky, clammy taste in the mouth; no appetite; but little thirst; feels nauseated if attempting to eat or drink; tenderness in the stomach and bowels; urine scant and high-colored and of a strong odor; stools sluggish and putrid; soreness in the joints and extremities; joints swollen and sore; sore in all of the extremities, aggravated by touch or motion; ameliorated by perfect rest, but restless; must move even if the pains are aggravated; restless, dreamy, worthless sleep until near daylight, when a quiet sleep is obtained, and when awakened is in a perspiration, and wants to curl down and go to sleep again; feels tired and does not want to be disturbed, but as soon as the perspiration drys up, which is soon after awakened, then the nervous restlessness returns; must move about, although the pains and soreness are greatly aggravated by every motion; the skin becomes dry and hot.

We will find every one of these symptoms under the pathogenesis of Byronia.

Let us now look at the mind symptoms. Gloomy and despondent; thinks every one has turned against them; thinks every one is talking about them or making sport of them or conspiring against them; would like to get out of sight and have a good cry all to themselves.

Would Byronia cure this case? If we give it we will most certainly meet with a failure, for the totality of the mind symptoms does not belong to that drug, and cannot cure it.

Again, vertigo; feels as if drunk; repeated attacks of fainting, congestive headache, heaviness in the head, vacant feeling in the head, confused feeling, with severe pain in the head, with pressure in the left side of the head; constant staring around at surrounding objects, buzzing in the ears, face cold and pale, stupid expression, putrid taste in the mouth, tonsils red, difficult swallowing, dread of water, empty eructations, cramps in the stomach and abdomen, sore to the touch; frequent urging to stool, with small discharges; urine scant and high-colored, constriction of the larynx, hysterical aphony, rattling breathing, dry, spasmodic, cough; tightness across the chest, pulse hard and strong, muscles of the chest sore, painful numbness of the hands, cramps in the thighs, trembling of the arms and hands, cold hands and feet, cries out when asleep, skin burning hot to the hand, heat, but no desire to drink. Now, here is a case in which every symptom is found in the pathogenesis of Hyoscyamus.

Let us look at the mind symptoms: Unconscious, sees ghosts, hears voices, sees strange objects, imagines that animals are jumping at them, converses with spirits, cannot bear to be alone, alternate exultations and melancholy, pangs of conscience, reeling, as if drunk.

Shall we give Hyoscyamus? Will it cure this case? It will most certainly fail, as it does not cover the mind symptoms, and would be worse than useless.

Vertigo from the use of narcotics, vertigo on rising from bed or a seat; worse after meals, grasps at the head; sensation of coldness in the cerebellum, sensation of stiffness in the brain, headache over the left eye, hot vertex, congestion to the head, with numbness in the limbs, worse in a warm room.

Scaly bald spots on the head, copious dandruff on the scalp, hair falls out, scratching makes the itching worse; after reading a dull pain deep in the eyes; black spots before the eyes, worse from looking at bright objects; contracted pupils, sounds reverberate in the ears, noises in the ears, with throbbing sensations; profuse green discharge of mucus from the nose, without coryza; sensation of fullness in the nose high up in the left nostril, with loose mucus; wing-like motion of the ali nasi, freckles on the nose, face ashy pale, circumscribed spots on the cheeks, puffed under eyes, nose, and lips; mouth and throat dry, no relief from water, gums stand off from the teeth and bleed easily, taste slimy and bitter, tongue coated whitish yellow, more in the middle; mucus in the throat removed with difficulty, sensation as of cotton in the throat, wants to eat, but as soon as food is offered does not want it; wants ice-cream or something refreshing, aversion to meats or sweets, regurgitation of food without nausea; as soon as water becomes warm in the stomach it is thrown up; gurgling in the stomach, with goneness in the region of the stomach; rolling and rumbling in the abdomen, with empty feeling; chronic painless undigested stools, with thirst for water during the night; urine scanty and frequent, with red sediment; frequent erections, with sexual weakness; larynx sensitive to the touch, larynx feels as if lined with fur, with roughness and rawness, worse in the evening; cough worse from a change of weather, especially from warm to cold; sputa grayish brown and tough, stitches through the left chest, congestion of the chest, worse from excitement or any emotion; burning in the palms, with clammy sweat; feet swollen in the evening; when walking makes missteps from weakness; limbs tremble from exertion, over-sensitive to external impressions, light noise, odors, or touch; sleepy in the day-time, but restless at night; feels stupid in the morning, as if they had had no sleep; tired, does not want to stir; profuse night-sweats, clammy, worse when asleep; rigors and heat alternate, brown spots in the skin of the chest and body.

All of these symptoms can be found in the pathogenesis of Phosphorus, and here we have a case of phthisis pulmonalis. Shall we give it?

Let us look at the mind symptoms. Must keep in motion day and night. Brain felt as if stirred up; felt as if going insane; melancholy mood, low spirited; irritable and sensitive, quarrelesome, flies into a passion at trifles, and when it has passed off feels like crying, low spirited, or extreme irritability.

Here we find that Phosphorus is not the remedy, and if given in high potency will most certainly kill the patient in a short time.

A great deal has been said about giving of Phosphorus to old consumptives, when it was indicated by the majority of the symptoms, and if it was given and injured the patient, then there was a fault in the law, and that it could not be universal. If they had considered the mental symptoms it would not have been given, as it was *not* the homœopathic remedy for the case, it only shows that if the wrong remedy is given even in a very high potency, it is not only superfluous but will do the case a great harm, hence Hahnemann was right in saying that we should pay particular attention to the mental symptoms of the patient, and also to those caused by the drug itself. I think that this explains why many cases are injured by carelessly examining the mental condition as Hahnemann has directed us to do in all examinations.

